# Multibaseline Interferometric Phase Denoising Based on Kurtosis in the NSST Domain

**DOI:** 10.3390/s20020551

**Published:** 2020-01-19

**Authors:** Yanfang Liu, Shiqiang Li, Heng Zhang

**Affiliations:** 1 School of Electronic, Electrical and Communication Engineering, Chinese Academy of Sciences, Beijing 100190, China; 2 Department of Space Microwave Remote Sensing System, Aerospace Information Research Institute, Chinese Academy of Sciences, Beijing 100190, China; lishq@mail.ie.ac.cn (S.L.); zhangheng@aircas.ac.cn (H.Z.)

**Keywords:** multibaseline interferometric synthetic aperture radar (InSAR), non-subsampled shearlet transform (NSST), kurtosis, noise level eatimation

## Abstract

Interferometric phase filtering is a crucial step in multibaseline interferometric synthetic aperture radar (InSAR). Current multibaseline interferometric phase filtering methods mostly follow methods of single-baseline InSAR and do not bring its data superiority into full play. The joint filtering of multibaseline InSAR based on statistics is proposed in this paper. We study and analyze the fourth-order statistical quantity of interferometric phase: kurtosis. An empirical assumption that the kurtosis of interferograms with different baselines keeps constant is proposed and is named as the baseline-invariant property of kurtosis in this paper. Some numerical experiments and rational analyses confirm its validity and universality. The noise level estimation of nature images is extended to multibaseline InSAR by dint of the baseline-invariant property of kurtosis. A filtering method based on the non-subsampled shearlet transform (NSST) and Wiener filter with estimated noise variance is proposed then. Firstly, multi-scaled and multi-directional coefficients of interferograms are obtained by NSST. Secondly, the noise variance is represented as the solution of a constrained non-convex optimization problem. A pre-thresholded Wiener filtering with estimated noise variance is employed for shrinking or zeroing NSST coefficients. Finally, the inverse NSST is utilized to obtain the filtered interferograms. Experiments on simulated and real data show that the proposed method has excellent comprehensive performance and is superior to conventional single-baseline filtering methods.

## 1. Introduction

Interferometric synthetic aperture radar is an important extension of synthetic aperture radar (SAR), which is extensively adopted to topography surveying [1], surface deformation monitoring [2] and so forth. Multi-baseline interferometry can comprehensively utilize the diversity of interferograms with different baselines in the same scene to effectively extract the height information of difficult topography, particularly under the circumstance in which the interferometric phase does not satisfy phase continuity assumption [3]. The interferometric phase filtering is a critical step in multibaseline interferometric SAR (InSAR). The interferometric phase is contaminated by massively coherent noise brought from thermal noise decoherence, baseline decoherence, time decoherence and many other decoherent factors in practice [4]. The noise directly affects the difficulty of subsequent phase estimation and the accuracy of the final digital elevation model (DEM). The main motivation of the interferometric phase filtering is to eliminate noises as much as possible while preserving most of the detail information.

As matters stand, the filtering methods of multibaseline InSAR are mainly divided into two categories. One applies the filtering method of single-baseline InSAR to denoise multiple interferograms separately. The filtering method of single-baseline InSAR is divided into two parts: the method in spatial domain and the method in transform domain. Some spatial filters, such as boxcar filter [5], Lee filter [6], local frequency estimate algorithm [7], optimal integration-based adaptive direction filter [8], and so forth, denoise along the gradient direction of interferometric phase. Differences between different methods are the process of detecting the direction and the weight of neighborhood pixels. The appearance of the nonlocal InSAR estimator (NL-InSAR) [9] makes the method in spatial domain reach a new stage. It simultaneously estimates the reflectivity, phase, coherence based on maximum likelihood estimation and the non-local similarity of interferograms. In addition, other methods based on non-local similarity have also been proposed successively [10]. What drives the outstanding performance of another part is the different characteristic between signal and noise in transform domain. It comprises of Goldstein method [11,12], wavelet filter [13,14], InSAR-BM3D [15], and so forth Wherein, Xu et al. applied the simultaneously sparse regularized reconstruction of amplitude and interferometric phase to acquire filtered interferograms [16]. InSAR-BM3D, which is the state of art method in transform domain, extends non-local block-matching 3-D (BM3D) to InSAR and reaches a great edge-Preserving performance. This kind of method does not put forward more requirements about the filtering process but focuses on improving the robustness of phase estimation. The filtering performance is not further improved.

Another category is the multibaseline joint filtering method. The strategy of multibaseline InSAR filtering methods is divided into two parts. One works on the SAR data stacks. NL-InSAR can be regarded as a special case of this part, and the number of SAR images is two. The filtered interferogram is extracted from the covariance matrix. And the covariance matrix is estimated with help of the average effect of statistically homogeneous pixels which have a similar statistical distribution with the central pixel [17,18,19,20]. The method can obtain despeckled amplitude images, coherence values, filtered interferograms simultaneously. But its performance is affected by the size of the data set. Large data sets are easy to obtain a more accurate estimation. Most methods require at least eight SAR images. Another one works on the InSAR data stacks, that is, a tensor composed of interferograms. In [21], You et al. proposed a tensor-based filter, which perceived the clean multibaseline InSAR data as a tensor with a low-rank matrix and drawn support from the Kronecker Basis Representation (KBR) to transform the filtering process into an estimation of a low-rank matrix. What demonstrates the potentiality of multibaseline joint filtering is that the method is superior to some state of the art single-baseline filter, for example, NL-InSAR, InSAR-BM3D, and so forth. But it still needs many interferograms to ensure the accuracy of the estimation [17,18].

This paper is an exploration of multibaseline interferometric phase filter based on the statistical characteristic. We propose a new filter on the basis of the NSST filter which is a part of the wavelet filter. The interferogram contains a large number of edges, fringes and other high-dimensional anisotropies. The NSST produces a multi-scaled and multi-directional sparse representation to images optimally and drives a more meticulous depiction of the high-dimensional anisotropies. The interferogram is decomposed into coefficient components with various scales and directions. the coefficient component involves little significant information with large amplitude and noise spreading in whole frequency domain. Coefficients, which are considered as noise are removed immediately, while the significant information is retained or shrinked. A pre-thresholded Wiener filter [22] is applied to eliminate noise. Then the inverse NSST is applied to obtain the reconstructed image.

The noise variance, which decides whether the coefficient is zeroed or retained, is a critical parameter of the Wiener filter. The accuracy of noise variance determines whether the performance of the wiener filter is optimal. A noise level estimation framework which is conceptually similar to the method in Reference [23] is proposed based on the kurtosis model in NSST domain and baseline-invariant property of kurtosis that is proposed and confirmed in this paper. The noise variance estimation is converted into a modified non-convex optimization problem. Moreover, the proposed estimator has higher operation efficiency due to skipping the clustering operation in Reference [23]. Considering the noise variance is space-variant, block estimation is applied. With the help of estimated noise variance, the wiener filter eliminates noise more accurately. Last but not least, the result of experiments on simulated data and real data confirms the efficiency and excellent performance of the proposed method.

## 2. Method

### 2.1. Signal Model

In the case of single-look, the probability density function of interferometric phase can be represented as (1). The interferometric phase satisfies additive noise model in spatial domain, which is deduced in Reference [13]. It can be expressed as (2).

(1) $$\begin{array}{cc} pdf(\phi ;\gamma ,\phi_{0})= & \frac{1}{2\pi }\frac{1-|\gamma^{2}|}{1-|\gamma^{2}|cos^{2}\left(\phi -\phi_{0}\right)}\\ \; & \;\cdot \;\left\{1+\frac{|\gamma |cos\left(\phi -\phi_{0}\right)cos^{-1}[-|\gamma |cos\left(\phi -\phi_{0}\right)])}{\left[1-\right|\gamma^{2}{\left|cos^{2}\left(\phi -\phi_{0}\right)\right]}^{1/2}}\right\},-\pi <\left(\phi -\phi_{0}\right)\leq \pi \end{array}$$

(2) y=x+n,

where *x* is the ideal phase deduced by the natural topography. *y* is the observed phase disturbed by the zero mean noise *n*, which is assumed to be independent of *x*. The phase jump ranged from −π to π, which is induced by the interferometric phase wrapping, derived a high frequency similarity to noise in frequency domain. Therefore, not surprisingly, it is apt to greatly be confused with noises. It is desirable that we convert the image to the complex domain to get the continuous complex phase and filter the real part and the imaginary part respectively. The signal model in the complex domain can be induced as

(3) exp(jy)=cos(y)+jsin(y).

The real part and the imaginary part can be expressed as

(4) $$cos\left(y\right)=N_{c}cos\left(x\right)+n_{c}$$

(5) $$sin\left(y\right)=N_{c}sin\left(x\right)+n_{s},$$

where $N_{c}=\frac{\pi }{4}|\gamma |F(\frac{1}{2},\frac{1}{2}{;2;|\gamma |}^{2})$ and $F(\frac{1}{2},\frac{1}{2};2;|\gamma {|}^{2})$ is the Gaussian hypergeometric distribution function. $n_{c}$ and $n_{s}$ are zero-mean random variables, which are generally assumed to additive Gaussian white noises in the filtering process.

### 2.2. Denoising Based on NSST

#### 2.2.1. The Nonsubsampled Shearlet Transform

Wavelet is prone to deal with 1-D signals existing pointwise singularities. Nevertheless, it is weak to handle multidimensional data dominated by distributed discontinuities, such as edges and fringes. In an effort to solve this problem, the wavelet basis with much higher directional sensitivity and more flexible shapes is encouraged for effectively capturing the singularity features of multidimensional data, involving composite wavelets [24], contourlets [25], and so forth. The shearlet transform is an important part of composite wavelet theory, which merges classical geometry and multiscale analysis [26,27,28,29]. The shearlet provides nearly optimal nonlinear approximation performance and produces an optimal sparse representation of images with distributed discontinuities. Thanks to its time-frequency local feature and directional sensitivity, the shearlet transform can be applied in image processing, for example, image denoising, image fusion, texture feature extraction, and so forth. In the context of composite wavelet, the discrete shearlet is defined as

(6) $$SH\left(\psi \right)=\left\{\psi_{j,l,k}=2^{\frac{3j}{2}}\psi \left(G^{l}S^{j}x-k\right):j\geq 0,-2^{j}\leq l\leq 2^{j},k\in Z^{2}\right\}$$

(7) $$S=\left(\begin{array}{cc} 4 & 0\\ 0 & 2 \end{array}\right),G=\left(\begin{array}{cc} 1 & 1\\ 0 & 1 \end{array}\right),$$

where $\psi \in L^{2}\left(R^{2}\right)$. *S* is the anisotropic dilation matrix related to scale transformation. *j* denotes the scale parameter in particular, which dominates the refinement of frequency and the redundancy of basis elements. *G* is the shear matrix related to geometrical transformation. *l* denotes the shear parameter which restricts the orientation of each shearlet element. Moreover, *k* indicates the shift parameter to locate distributed discontinuities in spatial domain. Calculating the Fourier transform to elements $\psi_{j,l,k}\left(x\right)$, we get

(8) $${\hat{\psi }}_{j,l,k}\left(w\right)=2^{-\frac{3j}{2}}\psi \left(wS^{-j}G^{-l}\right)e^{2\pi iwS^{-j}G^{-l}k}.$$

**Figure 1:**
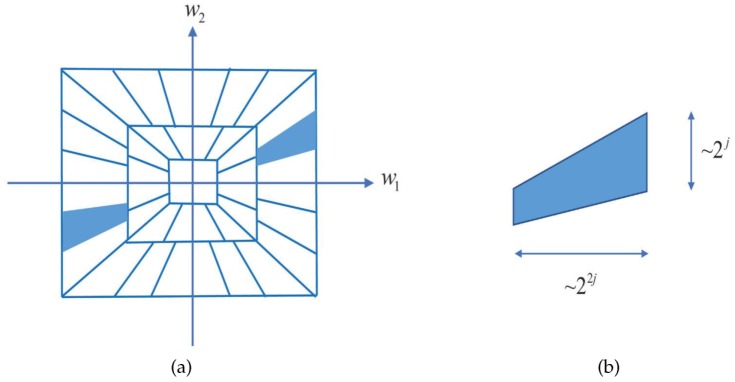
(**a**) The partition of frequency domain; (**b**) Frequency structure of the shearlet ${\hat{\psi }}_{j,l,k}\left(w_{1},w_{2}\right)$, for $w_{1}>0,w_{2}>0$.

It has frequency support as (9). The frequency division produced by the shearlet transform is illustrated in Figure 1.

(9) $$supp{\hat{\psi }}_{j,l,k}\subset \left\{\left(w_{1},w_{2}\right):w_{1}\in \left[-2^{2j-1},-2^{2j-4}\right]\cup \left[2^{2j-4},2^{2j-1}\right],\left|\frac{w_{2}}{w_{1}}-l2_{-j}\right|\leq 2^{-j}\right\}$$

The asymptotic approximation error of the shearlet transform is $N^{-2}{\left(logN\right)}^{3}$ when N→∞ [29]. So it precisely depicts the interferometric fringe. Besides, the shearlet forms Parseval frames in frequency domain. Its elements are trapezoidal pairs whose area is $2^{j}\times 2^{2j}$ and oriention is along the zero-crossing line with slope of $-l2^{-j}$ [29]. The corresponding orientation in spatial domain is along the line with slope of $l2^{-j}$. the shearlet elements can be discriminated by scales, locations and orientations. In addition, it apace decays in spatial domain. The aforementioned content indicates the highly directional sensitivity of shearlet, which makes a huge difference in the interferogram filtering.

**Figure 2:**
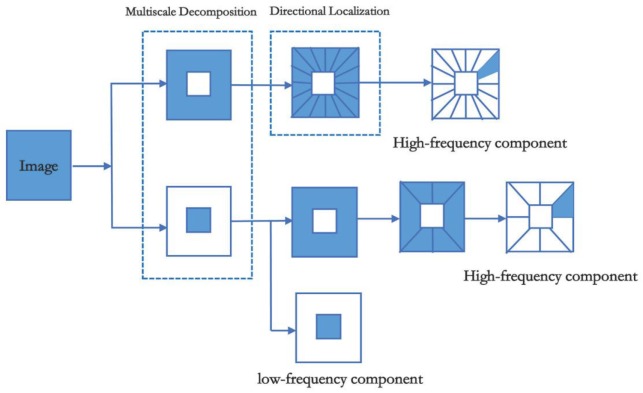
Decomposition process of non-subsampled shearlet transform (NSST).

In practice, the shearlet is shift-variant. The shearlet transform adopts the shift operation of the window function to realize the directional filtering. It involves a subsampling operation, which causes spectral aliasing in frequency domain. Thereby the Gibbs distortion occurs in the reconstructed image. To solve this problem, Easley and Labate proposed the nonsubsampled shearlet transform (NSST) which is enlightened by the great performance of the nonsubsampled contourlet transform. The NSST replaces the subsampled operation with convolution in the directional filtering. It is shift-invariant and efficiently eliminates the pseudo-Gibbs phenomenon in reconstructed images. Hereby the reconstructed image is more effective and intuitive. The decomposition procedure primarily contains two steps as shown in Figure 2.

#### Step 1: Multiscale Decomposition

The image is decomposed into a high-frequency component and a low-frequency component by means of non-subsampled pyramid (NSP). Then iteratively execute this step till image is decomposed into the *j* scales.

#### Step 2: Direction Localization

The core of direction localization is non-subsampled shearing filter banks (NSSFB), which impose the 2-D convolution of the shearing filter and the high-frequency component on the cartesian domain. The convolution averts subsampled operation, thereby the NSST is shift-invariant.

#### 2.2.2. Pre-Thresholded Wiener Filter

On account of the shift-invariant property, the NSST displays great performance in image denoising, particularly for the texture image. It is also desirable that the NSST filter exploits the coefficient shrinkage method which is consistent with the wavelet filter. Shearlet gives a sparse expression to images. That is to say, the intrinsical information of image is concentrated on few coefficients spreading over each scale with a considerable large amplitude. By contrast, shearlet coefficients generated by noise widely distribute in shearlet domain and its amplitude is small. Owing to this feature, a more accurate pre-thresholded Wiener filtering method with known noise variance is employed to remove the noise component. It consists of two steps:the pre-thresholded operation and Wiener filter. The pre-thresholded operation ensures the smaller local expected square error (LESE) of linear approximation, which is represented as

(10) $$\tilde{c}\left(i,j\right)=\left\{\begin{array}{cc} c(i,j) & \sigma_{c_{i,j}}^{2}>k\sigma_{n}^{2};\\ 0 & otherwise. \end{array}\right.$$

(11) $$k=1+\sqrt{\frac{2}{{\left(2N+1\right)}^{2}}}$$

(12) $$\sigma_{c_{i,j}}^{2}=\frac{1}{{\left(2N+1\right)}^{2}}\sum_{m,n=-N}^{N}c_{i-m,j-k}^{2}.$$

Then the Wiener filter obtains the best linear estimation of clean images. It is represented as

(13) $$\hat{c}\left(i,j\right)=a\left(i,j\right)\tilde{c}\left(i,j\right)$$

(14) $$a\left(i,j\right)=\frac{max(\sigma_{{\tilde{c}}_{i,j}}^{2}-\sigma_{n}^{2},0)}{\sigma_{{\tilde{c}}_{i,j}}^{2}}.$$

### 2.3. Noise Level Estimation Based on Kurtosis

The noise variance is the crux of the Wiener filter. The robust noise level estimator [30] designed by Donoho et al. regards scales median of absolute coefficients as noise variance and is commonly used in many papers. It is straightforward and expedient but tends to over-filter in interferograms with high signal-noise ratio (SNR). A more precise noise variance estimator is extremely urgent to improve the filtering performance of the NSST filter. The most primary innovation of this paper is to introduce the kurtosis-based noise level estimator in Reference [23] to multibaseline InSAR. The kurtosis and the noise variance have a certain relationship in the additive Gaussian white noise model. There exist two unknown variables in the kurtosis model, the number of unknown variables is larger than the number of equations. The result of the minimization method, such as l1-minimization [31], l2-minimization [32] and so forth, exists great errors. To solve the problem, Dong et al combine the kurtosis model with a constraint, in which the kurtosis of images with different structures or statistical behaviors should be unequal, to improve the estimation accuracy of the noise variance. The K-means clustering process is applied to classify the whole image into non-overlapping image patches with different structures.

In this section, the kurtosis of the interferometric phase is introduced. And a special property of the kurtosis is proposed in multibaseline InSAR and is named as the baseline-invariant property. Along with the idea in Reference [23], the baseline-invariant property is regarded as a constraint to ensure the accurate estimation of the noise level. The modified method omits the clustering process and eliminates errors introduced by the fault of the cluster. Efficiency and performance get promoted. Next, the noise level estimator is introduced in two parts. The first one introduces the kurtosis of the interferometric phase and two important properties of the kurtosis. In the other part, the noise level estimation is introduced in detail.

#### 2.3.1. Kurtosis

**Figure 3:**
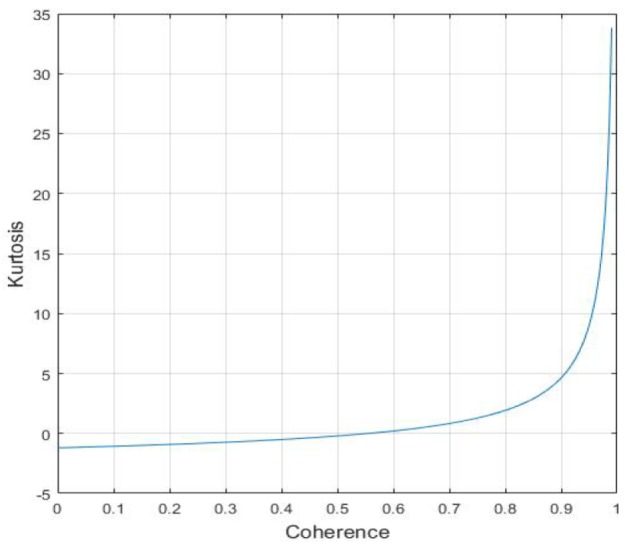
The kurtosis of interferometric phase.

The image is decomposed into various coefficient components at different scales and directions by NSST. The research on the distribution of NSST coefficient components is conducive to the further analysis of images and is a significant topic in image processing. Among them, the research on its statistics is of great potential. The low-order statistic is weak, even invalid in interferograms which involve a large amount of textures and detail informations. Consequently, the scholar begins with the study of its higher-order statistic, such as kurtosis and skewness. In this section, we study and analyse the kurtosis of interferograms and NSST coefficient components. The kurtosis of a random variable *Y* is defined as

(15) $$\kappa \left(Y\right)=\frac{C_{4}\left(Y\right)}{C_{2}^{2}\left(Y\right)}-3,$$

where $C_{k}\left(•\right)$ is the kth cumulant function. The kurtosis reveals the concentration level of the probability density function. Intuitively, the kurtosis reflects the sharpness of the probability density distribution, wherein the kurtosis of the Gaussian distribution is 0. Based on the single-look probability density function of the interferometric phase, the kurtosis is calculated numerically as a function of the coherence, as shown in Figure 3. It indicates that the kurtosis is proportional to the coherence. Obviously, the clean interferogram emerges higher kurtosis when compared with interferograms disturbed by coherent noise with variable degrees. When the noise is strong enough to destroy the fringe structure of interferograms, the kurtosis is smaller due to the influence of the noise. In addition, the kurtosis tends to a negative number when the coherence is close to zero owing to the impact of non-Gaussian noises. In contrast, when the coherence is high, the fringe structure of interferograms plays a primary role. So the kurtosis increases with the improvement of coherence. It should be noted that NSST coefficient components of interferograms are sparse and its kurtosis is greater than zero.

The proposed method takes advantage of two vital properties of the kurtosis of interferograms. One is the scale-invariant property, which works well on all natural images, that is, the kurtosis of coefficient components in the Linear Transform Domain should be held constant at different scales. It is verified and revised by some work in References [31,32,33]. A modified description for the scale-invariant kurtosis assumption is that the stability is effective in clean images throughout all scales and the variation is the specific impact of noise [32]. The scale-invariant property in the nonsubsampled shearlet transform can be formalized as (16), where $Y_{i}$ is the ith NSST coefficient component of the clean image *Y*.

(16) $$\kappa \left(Y_{i}\right)=\kappa \left(Y_{j}\right),i,j=1,2,\ldots ,N.$$

Another property, which is particular to interferograms and can be yielded from an empirical summary, is proposed in this paper. The kurtosis of images with similar structure or statistical behavior is assumed to be a constant [23]. Along this line, we suggest that the kurtosis of interferograms with different baseline keeps constant and denote it as the baseline-invariant property. Then an interpretation from two perspectives should be introduced. First, It will be explicated further in terms of the probability density function (pdf). The pdf of the interferometric phase is independent of baselines, so does the kurtosis. It can be revealed in (1). In other words, the kurtosis of the interferometric phase is baseline-invariant. However, in virtue of impacts of discretization operation such as sampling, numerical calculation and so forth, the kurtosis of interferograms with different baseline fluctuates around a constant in reality. Fortunately, the fluctuation variance is small enough. So the negative effect of the fluctuation variance can be ignored. On the other hand, as far as the image is concerned, interferograms of the same topography with different baseline intuitively have similar texture trends which show similar statistical behaviors. Correspondingly, the kurtosis maintains invariant in images with similar statistical behaviors, which gives strong support for the baseline-invariant property of kurtosis in multibaseline InSAR.

**Figure 4:**
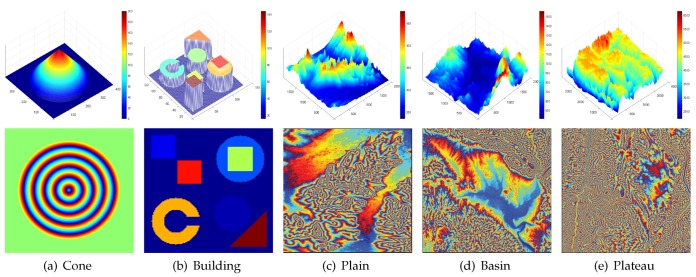
Five different topography (top) and their typical interferogram (bottom): (**a**)–(**e**) represent cone, building, plain, basin and plateau respectively.

Some simulated analyses prove the baseline-invariant property. In order to verify the validity of that property for various types of interferograms (generated by various topographies), we select the DEM of five common topography, including Cone, Building, the Northeast plain, China (the elevation below 500 m, the relief is not more than 200 m), the Sichuan Basin, China, Mangkang Mountain, Tibet, China (Plateau, the elevation above 500 m, the relief is more than 200 m). All primordial elevation data are derived from simulated data and Shuttle Radar Topography Mission DEM (SRTM-DEM) elevation data which is provided by Geospatial Data Cloud site, Computer Network Information Center, Chinese Academy of Sciences (http://www.gscloud.cn). The elevation data and typical interferograms of them are shown in Figure 4.

In the light of the DEM of ground scenes and parameters of multibaseline InSAR simulation systems as shown in Table 1, we start with projecting the elevation data into the slant coordinate system. Then we calculate 81 ideal interferograms for each topography when the baseline varies from 50 m to 500 m based on the interferometry principle.

**Figure 5:**
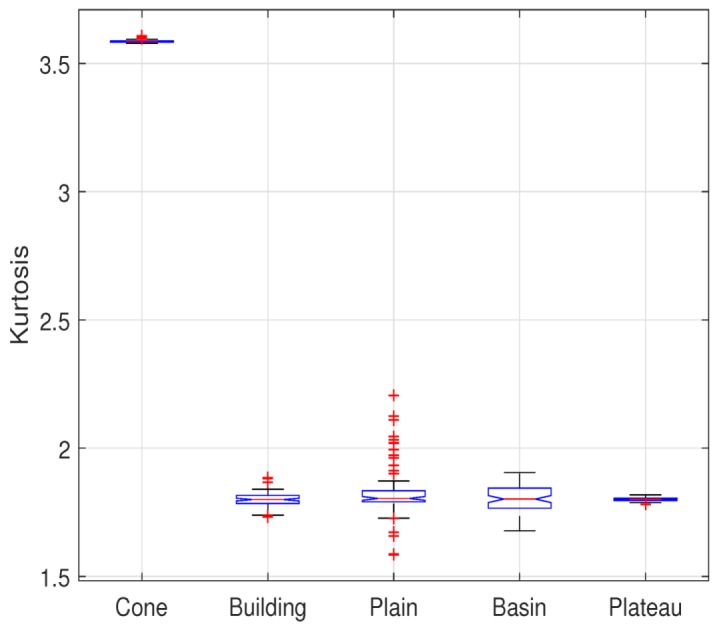
Boxplot of kurtosis corresponding to various topography.

The kurtosis of interferograms with different baseline corresponding to each topography is calculated and its boxplot is shown in Figure 5. In order to approach to the actual situation, the fringe density of simulated interferograms should not be too sparse or too dense. Therefore, the system central frequency for different topography is different. However, in this section, the baseline-invariant property of the kurtosis of interferograms with different baselineswe is confirmed. In other words, the fact that the kurtosis of interferograms with different fringe density remains constant is verified. So the change of the central frequency is not taken into account. We observe the kurtosis standard deviation of each topography numerically in Table 2. The result implies that the maximum standard deviation of kurtosis is 0.1056. Considered the influence of numerical calculation and sampling, it is interpreted that the kurtosis of interferograms with different baseline keeps constant.

#### 2.3.2. Noise Level Estimation

In this section, the principle and process of the noise level estimator proposed in this paper is introduced in detail. The real part and the imaginary part of interferograms are handled respectively. Taking the real part as an example, it is decomposed into *M* components in NSST domain. In addition, the additive noise model applies to all shearlet components.

(17) $$y_{i}=x_{i}+n_{i}$$

where $y_{i}$,$x_{i}$,$n_{i}$ represents the ith NSST coefficient of the observed phase, ideal phase and noise, respectively. The variance of $y_{i}$ is represented as

(18) $$\sigma_{y_{i}}^{2}=\sigma_{x_{i}}^{2}+\sigma_{n_{i}}^{2}$$

(19) $$\sigma_{n_{i}}^{2}=\sigma_{n}^{2}\;\cdot \;\sigma_{\lambda_{i}}^{2}$$

(20) $$C_{4}\left(y_{i}\right)=C_{4}\left(x_{i}\right)+C_{4}\left(n_{i}\right),$$

where $\sigma_{y_{i}}^{2}$, $\sigma_{x_{i}}^{2}$, $\sigma_{n_{i}}^{2}$ is the variance of $y_{i}$, $x_{i}$, $n_{i}$, respectively. $\sigma_{\lambda_{i}}^{2}$ indicates the estimated noise level of the ith NSST coefficient for a white Gaussian noise of standard deviation 1. It is calculated by the Monte Carlo Estimation Method. Then we deduce (21) from (18), (19) and (20):

(21) $$\sigma_{y_{i}}^{4}\kappa \left(y_{i}\right)=\sigma_{x_{i}}^{4}\kappa \left(x_{i}\right)+\sigma_{n_{i}}^{4}\kappa \left(n_{i}\right).$$

Since the assumption that $n_{i}$ obeys the Gaussian distribution, $\kappa (n_{i})=0$. Besides, the coefficient distribution of subband components is generally more centralized than the Gauss distribution, that is, $\kappa \left(x_{i}\right),\kappa \left(y_{i}\right)\geq 0$, because the interferogram is subdivided into subband components with different scales and directions. The deterministic relationship between noise variance and kurtosis is deduced from (18) and (21), as shown in (22).

(22) $$\sqrt{\kappa (y_{i})}=\sqrt{\kappa (x_{i})}-\frac{\sigma_{n}^{2}\;\cdot \;\sigma_{\lambda_{i}}^{2}}{\sigma_{y_{i}}^{2}}\sqrt{\kappa (x_{i})}.$$

Equation (22) is the kurtosis model which describes the deterministic relationship between the kurtosis and the noise variance. The kurtosis and variance of the observed phase $y_{i}$ can be calculated directly but the kurtosis of the ideal phase and the noise variance are unknown. The number of unknown variables is larger than the number of equations, so the noise variance cannot be determined directly by Equation (22). A large number of texture structures appearing in interferograms represent similar characteristics with noise in the frequency domain. The existence of texture structure leads to great errors of the noise variance estimation achieved by the minimization method of (22), that is, l1-minimization [31], l2-minimization [32]. To solve this problem, Equation (22) and the baseline-invariant property which acts as another equation are used to jointly estimate the noise variance. With the help of the new information from the baseline-invariant property, the estimation with higher accuracy is realized. the baseline-invariant property is represented as

(23) $$\sqrt{\kappa (x^{k})}=\sqrt{\kappa (x^{l})},k,l=1,2,\ldots ,N.$$

The form of sqrt is adopted for the convenience of the subsequent solution of optimization model. The following optimization model is proposed from (22) and (23).

(24) $$\begin{array}{cc} \left\{{\hat{\sigma }}_{n}^{2},{\left\{\hat{\kappa }\left(x^{j}\right)\right\}}_{j=1}^{N}\right\}= & \underset{{\hat{\sigma }}_{n}^{2},{\left\{\hat{\kappa }\left(x^{i}\right)\right\}}_{i=1}^{N}}{\arg\min}\{\sum_{k=1}^{N}\sum_{l=1}^{N}{\left(\sqrt{\kappa (x^{k})}-\sqrt{\kappa (x^{l})}\right)}^{2}\\ \; & +\sum_{j=1}^{N}\sum_{i=1}^{M}{\left(\sqrt{\kappa (y_{i}^{j})}-\sqrt{\kappa (x^{j})}+\frac{\sigma_{n}^{2}\;\cdot \;\sigma_{\lambda_{i}}^{2}}{\sigma_{y_{i}^{j}}^{2}}\sqrt{\kappa (x^{j})}\right)}^{2}\}\\ \; & subject\;to:\kappa \left(x^{j}\right)\leq \frac{1}{M}\sum_{i=1}^{M}\kappa \left(y_{i}^{j}\right),for\;j=1,2,3,\ldots ,N, \end{array}$$

where the superscript *j* denotes the jth baseline and the subscript *i* denotes the ith coefficient component. The first term of optimization function is deduced by the baseline-invariant property of kurtosis and another one is added for fitting the kurtosis model in (22). Then the constraint is derived from the fact that the kurtosis of coefficients decreases owing to the noise disturbance as shown in Figure 3.

The aforementioned optimization function is constrained and non-convex optimization problem with two variables: $\sigma_{n}^{2}$ and ${\kappa (x^{j})}_{j=1}^{N}$, which should be considered and optimized simultaneously. This constrained and non-convex optimization problem can be decomposed into two continuous and convex optimization sub-problems by fixing one variable to optimize another variable. Firstly, fix the noise variance $\sigma_{n}^{2}$ and then update ${\kappa (x^{j})}_{j=1}^{N}$. The optimization model 1 to be solved is

(25) $$\begin{array}{cc} {\left\{\kappa (x^{j})\right\}}_{j=1}^{N}= & \arg\min\{\sum_{k=1}^{N}\sum_{l=1}^{N}{\left(\sqrt{\kappa (x^{k})}-\sqrt{\kappa (x^{l})}\right)}^{2}\\ \; & +\sum_{j=1}^{N}\sum_{i=1}^{M}{\left(\sqrt{\kappa (y_{i}^{j})}-\sqrt{\kappa (x^{j})}+\frac{{\left({\hat{\sigma }}_{n}^{2}\;\cdot \;\sigma_{\lambda_{i}}^{2}\right)}_{t}}{\sigma_{y_{i}}^{2}}\sqrt{\kappa (x^{i})}\right)}^{2}\}\\ \; & =\arg\min\{\sum_{k=1}^{N}\sum_{l=1}^{N}{\left(\sqrt{\kappa (x^{k})}-\sqrt{\kappa (x^{l})}\right)}^{2}+\sum_{j=1}^{N}\sum_{i=1}^{M}\kappa \left(y_{i}^{j}\right)\\ \; & +2\sum_{j=1}^{N}\sum_{i=1}^{M}\left[\sqrt{\kappa (y_{i}^{j})}\left(\frac{{\left({\hat{\sigma }}_{n}^{2}\;\cdot \;\sigma_{\lambda_{i}}^{2}\right)}_{t}}{\sigma_{y_{i}}^{2}}-1\right)\sqrt{\kappa (x^{j})}\right]+\sum_{j=1}^{N}\sum_{i=1}^{M}{\left(\frac{{\left({\hat{\sigma }}_{n}^{2}\;\cdot \;\sigma_{\lambda_{i}}^{2}\right)}_{t}}{\sigma_{y_{i}}^{2}}-1\right)}^{2}\kappa \left(x^{j}\right)\}. \end{array}$$

Ignoring the second item which is independent with ${\kappa (x^{j})}_{j=1}^{N}$. Let

- the vector $k\in R^{N}$
  $$k={\left[\sqrt{\kappa (x^{1})},\sqrt{\kappa (x^{2})},\ldots ,\sqrt{\kappa (x^{N})}\right]}^{T}$$
- *A* is a diagonal matrix of N×N and the diagonal element is
  $$A_{ii}=\sum_{i=0}^{M}{\left(\frac{{\left({\hat{\sigma }}_{n}^{2}\;\cdot \;\sigma_{\lambda_{i}}^{2}\right)}_{t}}{\sigma_{y_{i}}^{2}}-1\right)}^{2}$$
- *B* is a symmetric matrix
  $$B_{ij}=\left\{\begin{array}{cc} N-1 & i=j;\\ -1 & otherwise. \end{array}\right.$$
- the vector $C\in R^{N}$
  $$c_{i}=\sum_{i=0}^{M}2\sqrt{\kappa (y_{i}^{j})}\left(\frac{{\left({\hat{\sigma }}_{n}^{2}\;\cdot \;\sigma_{\lambda_{i}}^{2}\right)}_{t}}{\sigma_{y_{i}}^{2}}-1\right).$$

Then the optimization function can be simplified as

(26) $$\arg\min\;k^{T}\left(A+B\right)k+c^{T}k.$$

Because A+B is a positive definite matrix, it is a standard convex optimization for quadratic programming with constraints and can be solved directly.

Similarly, fix ${\kappa (x^{j})}_{j=1}^{N}$ and update $\sigma_{n}^{2}$, we deduce optimization model 2 as shown in (27).

(27) $${\left({\hat{\sigma }}_{n}^{2}\right)}_{t+1}=\underset{{\left\{\kappa (x^{i})\right\}}_{j=1}^{N}}{\arg\min}\sum_{j=1}^{N}\sum_{i=1}^{M}{\left(\sqrt{\kappa (y_{i}^{j})}-\sqrt{{\hat{\kappa }}_{t}\left(x^{j}\right)}+\frac{\sigma_{n}^{2}\;\cdot \;\sigma_{\lambda_{i}}^{2}}{\sigma_{y_{i}}^{2}}\sqrt{{\hat{\kappa }}_{t}\left(x^{j}\right)}\right)}^{2}.$$

Let the partial derivative of function (27) equals to 0 and then we get the noise variance.

$$2\sum_{j=1}^{N}\sum_{i=1}^{M}\left(\sqrt{\kappa (y_{i}^{j})}-\sqrt{\hat{\kappa }\left(x^{j}\right)}+\frac{\sigma_{n}^{2}\;\cdot \;\sigma_{\lambda_{i}}^{2}}{\sigma_{y_{i}^{j}}^{2}}\sqrt{{\hat{\kappa }}_{t}\left(x^{j}\right)}\right)\frac{\sqrt{{\hat{\kappa }}_{t}\left(x^{j}\right)}}{\sigma_{y_{i}^{j}}^{2}}=0$$

(28) $${\hat{\sigma }}_{n}^{2}=\frac{\sum_{ij}\left(\sqrt{{\hat{\kappa }}_{t}\left(x^{j}\right)}-\sqrt{\kappa (y_{i}^{j})}\right)}{\sigma_{\lambda_{i}}^{2}\;\cdot \;\sum_{ij}\frac{\sqrt{{\hat{\kappa }}_{t}\left(x^{j}\right)}}{\sigma_{y_{i}^{j}}^{2}}}.$$

Iteratively update ${\kappa (x^{j})}_{j=1}^{N}$ and $\sigma_{n}^{2}$, until convergence.

A pivotal assumption of the aforementioned method is that the noise variance remains constant spatially. Namely, the noise is homogeneous throughout the image space. Yet the interferogram suffers the coherent noise with spatially variable characteristic. The consistent noise variance induces unbalanced filtering results. So we further advance the global noise variance to the local noise variance. Specifically, we divide the image into a certain amount of non-overlapping patches with the same size and assume the stability of noise variance in each patch and estimate its local noise variance simultaneously. The noise level estimation procedure can be summarized in Algorithm 1.
**Algorithm 1** Estimating the local noise variance ${\left\{{\left({\hat{\sigma }}_{n}^{2}\right)}_{l}\right\}}_{l=1}^{L}$**Input:**N×M NSST coefficients ${\left\{Y_{j}^{i}\right\}}_{i=1,j=1}^{M,N}$ of the observed interferograms with *N* different baselines, the size of patch m×n and the maximum iteration number $N_{iter}$.**Initialization:**${\left\{{\left({\hat{\sigma }}_{n}^{2}\right)}_{l}\right\}}_{l=1}^{L}$ = 0.**1:** Divide all coefficients into *L* patches whose size is m×n and calculate the kurtosis ${\left\{\kappa_{l}\left(y_{i}^{j}\right)\right\}}_{i=1,j=1,l=1}^{N,M,L}$ and variance ${\left\{{\left(\sigma_{y_{i}^{j}}^{2}\right)}_{l}\right\}}_{i=1,j=1,l=1}^{N,M,L}$ of eath patch.**2:** Repeat.**3:** Let ${\left\{{\left(\sigma_{n}^{2}\right)}_{l}\right\}}_{l=1}^{L}$ equals the solution of the last optimization, update ${\left\{{\hat{\kappa }}_{l}\left(x\right)\right\}}_{l=1}^{L}$ by optimization function 1.**4:** Let ${\left\{\kappa_{l}\left(x\right)\right\}}_{l=1}^{L}$ equals the solution of the step 3, update ${\left\{{\left({\hat{\sigma }}_{n}^{2}\right)}_{l}\right\}}_{l=1}^{L}$ by optimization function 2.**5:** Until ${\left\{{\left({\hat{\sigma }}_{n}^{2}\right)}_{l}\right\}}_{l=1}^{L}$ and ${\left\{{\hat{\kappa }}_{l}\left(x\right)\right\}}_{l=1}^{L}$ converges or $N_{iter}$ is reached.**6:** Return ${\left\{{\left({\hat{\sigma }}_{n}^{2}\right)}_{l}\right\}}_{l=1}^{L}$.

## 3. Results

In this section, we validate the efficiency and validity of the proposed method via extensive experiments on simulated and real interferograms. Experiments consist of three parts. First of all, we demonstrate the estimation accuracy of noise level on simulated noisy interferograms. It is the crux of the proposed method. Then, the simulated experiments are conducted. As a promotion of NSST filter, the developed method is compared with five state of the art single-baseline filters, including: Goldstein method, local frequency estimate (LFE) algorithm, optimal integration-based adaptive direction filter (OADF), iterative NL-InSAR and InSAR-BM3D. Finally, the proposed method on real interferograms will be tested. For simplicity, the proposed method is termed as NSST in the following sections.The parameters of various filters are set as

- Goldstein: the filtering window size is 32×32, α equals 0.9;
- OADF: the filtering window size is 7×7;
- LFE: the local frequency estimation window and filtering window are set to 9×9;
- NL-InSAR: the iterative number is 10;
- InSAR-BM3D: the parameters are consistent with [15];
- NSST: the decomposition scale equals 5. Each scale contains 16 different directions.

### 3.1. Noise Estimation Experiments

**Figure 6:**
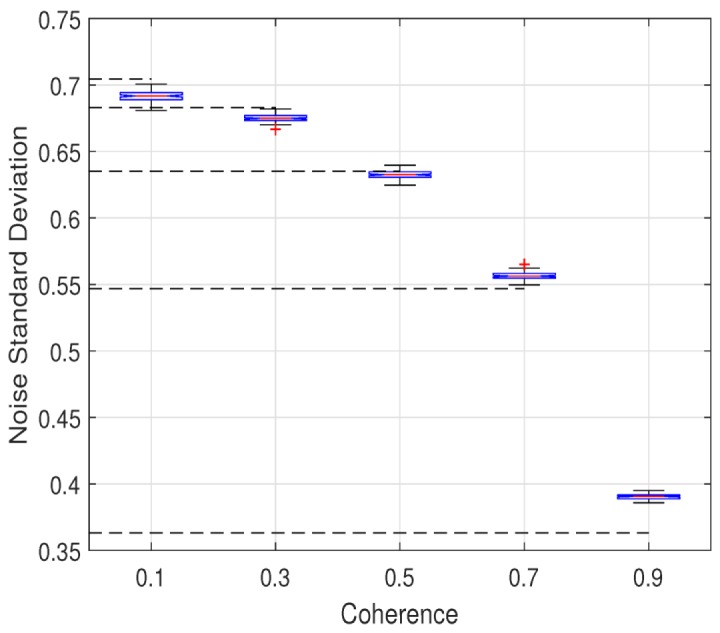
Boxplot of 100 noise level estimation experiments corresponding to each coherence (the black dotted line is the mean of true value).

The key of the proposed method is noise variance estimation. In this section, we verify the accuracy of the estimated noise variance on simulated data. The original elevation model is a cone, as shown in Figure 4a. As noted before, the interferometric phase accords with the additive noise model in complex domain, the real part and the imaginary part are denoised respectively. We generate three clean interferograms with different baseline. Noisy interferograms are disturbed by the circular complex standard Gaussian noise. The coherence of noisy interferograms is set to 0.1, 0.3, 0.5, 0.7, 0.9, respectively. The true noise variance of the real part, for example, is calculated numerically. Compared with the true value, the estimated noise variance is generated by the proposed method. In order to further test the robustness of the proposed method, 100 Mont-Calo simulations are conducted. Statistics of its results are shown in Figure 6, where the black dotted line is the mean of true value in 100 experiments.

The comparison between the estimation and the mean implies the accuracy and stability of the proposed method. The maximum error rate is calculated to evaluate the estimation accuracy and is defined as

(29) $$R_{M}=\frac{max(|\hat{\sigma }-\bar{\sigma }|)}{\bar{\sigma }}\times 100$$

where $\hat{\sigma }$ denotes the estimated value in 100 experiments, $\bar{\sigma }$ indicates the mean of true value. The result is shown in Table 3. It is obvious that some errors exist in the estimation. The higher the coherence is, the larger the SNR is. In the case of low coherence, the significant noise level engenders the confusion of the high-frequency information and the noise, which results in a slight overestimation. On the contrary, the noise near fringe in interferograms is mistaken for significant pixels owing to its weak effect to fringes in the case of high coherence. So the estimation is lower than the true value. We must emphasize that the maximum error rate is controlled within 8.76%. The underestimation is compensated by the excellent performance of Wiener filter.

### 3.2. Simulated Experiments

**Figure 7:**
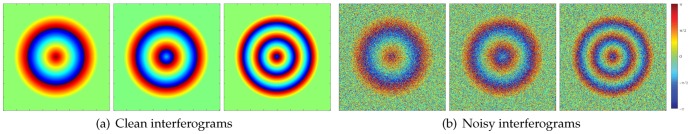
Clean interferograms and noisy interferograms generated by a cone with coherence of 0.5.

**Figure 8:**
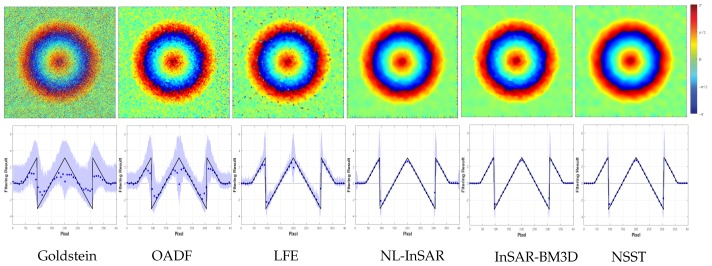
The filter results of the interferogram generated by a cone with coherence of 0.5 (top) and the statistical result of pixels at the center row (bottom, the black solid line is the true value; the blue dotted line denotes the mean of 100 experiments; the pale blue shadow is the range of three times standard deviation near the mean.).

In this section, we simulate three interferograms of cone and mountain to assess the performance of the proposed method. The noise environment comprises two situations: constant and variable noise variance in spatial domain. It is necessary for comparative experiments within each section. The experiments in interferograms with unitary noise variance are conducted to inspect the reconstructed performance for phase jump and phase gradient mutation. We select interferograms with 400×400 pixels, which are generated by cone and contain both phase jump and phase gradient mutation. Its clean interferograms and noisy interferograms can be shown in Figure 7. Coherence is set to 0.5. The block operation is omitted because of the constant noise variance. The comparable results of the interferogram with the shortest baseline are shown in the first row of Figure 8.

Intuitively, the result of the Goldstein method is incorrect. There are obvious errors in OADF and LFE. NL-InSAR, InSAR-BM3D and the proposed method all obtain appreciable results. The mean square error (MSE) between the clean interferogram and the filtered interferogram confirms above statements. What is more, Table 4 lists the number of residues in the filtered interferogram and the computation time. Note that the bold font indicates the best performance in the table. Table 4 exhibits that the proposed method outperforms to others with a running time that is second only to Goldstein method. The similar MSE are found in InSAR-BM3D but its computation time is about twice as long as our method. The results in NL-InSAR is superior to Goldstein method, OADF, LFE but its operation efficiency is the worst due to iterative operation. By and large, a combination of minimum MSE, minimum number of residues and high efficiency has taken in our method.

The second row of Figure 8 displays the mean and standard deviation of 100 Monte Carlo experiments at the central row of results. Thereinto, the black solid line is the true value. The blue dotted line denotes the mean of 100 experiments. The pale blue shadow is the range of three times standard deviation near the mean. The poorest result in Goldstein method is interrelated with fixed α and its boundedness to lower SNR. The result of NL-InSAR, InSAR-BM3D and our method for the stationary phase is close to unbiased estimation, while other methods emerge distinct deviation. The basic idea of OADF and LFE is the estimation to local direction and frequency of interferometric phase. So the invalid estimation has contributed to a heavy fluctuation near phase jump and phase gradient mutation. NL-InSAR produces excellent performance in phase jump but its non-local mean operation induces the outlier which can be observed on both sides. Nevertheless, it produces excellent performance in phase jump. Generally, InSAR-BM3D and our method outperform other methods but our method has higher operation efficiency.

**Figure 9:**
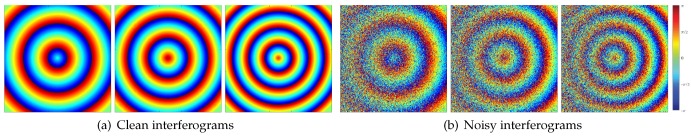
Clean interferograms and noisy interferograms generated by a cone with coherence ranging from 0.1 to 0.9.

Considering a more complex noise level model in the second experiment, in which the coherence ranges from 0.1 to 0.9 and increases from left to right at regular intervals. Figure 9 shows clean interferograms and noisy interferograms with the size of 240×240. The image is divided into 9 non-overlapping patches in noise variance estimation procedure. The size of each patch is 80×80.

In this part, a new evaluation index, which is expressed as the pixel-wise Gradient Magnitude Similarity (GMS) [34,35] between the reference and filtered images, is adhibited to evaluate the filtering results of various methods. Gradient magnitude is an apparent indication of the difference between adjacent pixels. The gradient of interferometric phase consists of two parts: the gradient of the local stationary phase reflects the local slope of topography and the similarity of gradient casts light upon the similarity of local topography. In addition, the outlier implies phase discontinuity within a phase period. Similar to the well-known Structure SIMilarity (SSIM) index, the gradient similarity of phase jump can also reflect the edge-preserving ability of methods. Therefore, it is worth to use GMS as a new evaluation index. GMS is defined as

(30) $$GMS\left(i\right)=\frac{2G_{o}\left(i\right)G_{f}\left(i\right)+\lambda }{G_{o}^{2}\left(i\right)+G_{f}^{2}\left(i\right)+\lambda },$$

where λ is set to 0.0026 to ensure numerical stability. $G_{o}$ and $G_{f}$ indicate the gradient magnitudes of *o* and *f*. The gradient magnitudes is derived from (31) and (32).

(31) $$G_{o}\left(i\right)=\sqrt{{\left(o⊗h_{x}\right)}^{2}+{\left(o⊗h_{y}\right)}^{2}}$$

(32) $$G_{f}\left(i\right)=\sqrt{{\left(f⊗h_{x}\right)}^{2}+{\left(f⊗h_{y}\right)}^{2}},$$

where *o* and *f* indicate the original images and filtered images respectively. $h_{x}$ and $h_{y}$ indicate the Prewitt filter along the horizontal and vertical direction. They are derived from (33).

(33) $$h_{x}=\left[\begin{array}{ccc} \frac{1}{3} & 0 & -\frac{1}{3}\\ \frac{1}{3} & 0 & -\frac{1}{3}\\ \frac{1}{3} & 0 & -\frac{1}{3} \end{array}\right],h_{y}=\left[\begin{array}{ccc} \frac{1}{3} & \frac{1}{3} & \frac{1}{3}\\ 0 & 0 & 0\\ -\frac{1}{3} & -\frac{1}{3} & -\frac{1}{3} \end{array}\right]$$

It should be noted that the larger the GMS value is, the higher the quality of the restored image is. When GMS=1, the reference image is fully recovered. The mean of GMS map (GMSM) refers to the overall performance of GMS map.

**Figure 10:**
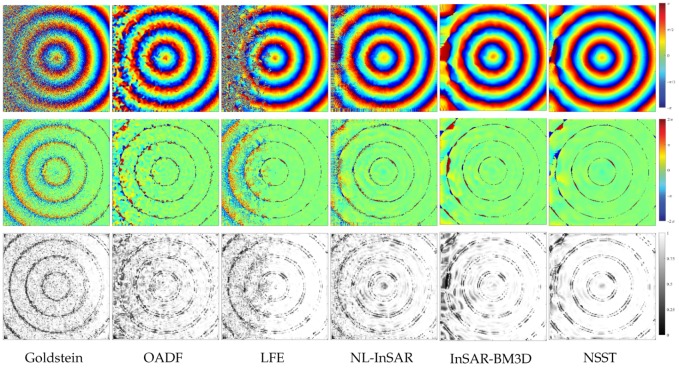
The filter results of the interferogram generated by a cone with variable coherence (**top**), the residuals graph (**middle**) and the Gradient Magnitude Similarity (GMS) map (**bottom**).

Figure 10 shows the filtering results, residual graph and GMS map corresponding to six different filters. Results show that all methods can correctly restore the original phase in the high-coherence region. However, only InSAR-BM3D and the proposed method get considerable results in the low coherence region. Besides, as far as the GMS map is concerned, our method has better ability to maintain the phase gradient, especially in the low-coherence region. The estimated phase of the proposed method tends to be more stationary and closes to the original phase. Table 5 shows the MSE, GMSM and computation time. Note that the bold font indicates the best performance in the table. The performance of various methods can be expressed as (where > denotes better performance):

- MSE: NSST>InSAR-BM3D>NL-InSAR>OADF>LFE>Goldstein
- GMSM: NSST>InSAR-BM3D>NL-InSAR≥LFE>OADF>Goldstein
- Computation efficiency: Goldstein>NSST>InSAR-BM3D>OADF>LFE>NL-InSAR

As a whole, our method is superior to other methods.

**Figure 11:**
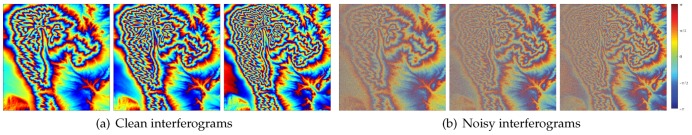
Clean interferograms and noisy interferograms generated by a mountain with coherence ranging from 0.1 to 0.9.

We consider a more complex topography to test the performance of various methods. The elevation data of a steep mountain in Shaanxi Province, China is selected to generate three interferograms. Coherence is consistent with last experiment. The size of interferograms is 1600×1600. The image is divided into 25 non-overlapping patches in noise variance estimation procedure. The size of each patch is 320×320. Interferograms involve dense and sparse fringes. Dense fringes are mostly located in the region with low coherence, which can better verify the effectiveness of the proposed method. Figure 11 shows clean interferograms and noisy interferograms.

**Figure 12:**
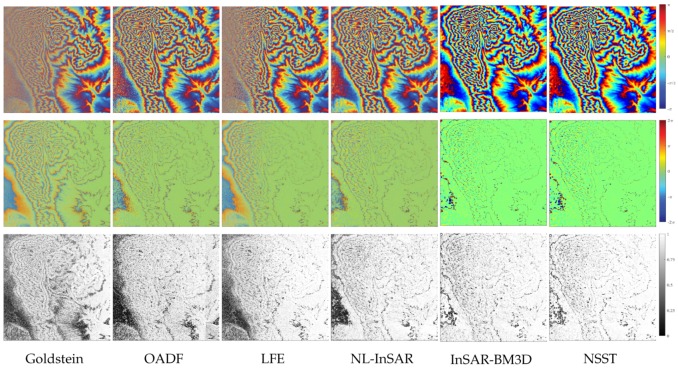
The filter results of the interferogram generated by a complex topography with variable coherence (**top**), the residuals graph (**middle**) and the GMS map (**bottom**).

Figure 12 shows the filtered results. Table 6 shows the results are similar to the results of the previous experiment. Note that the bold font indicates the best performance in the table. The proposed method produces minimum MSE and maximum GMSM, which prove the prominent filtering performance of it. The minimum MSE of the proposed method proves that the result of the proposed method is closer to the true interferometric phase. And the maximum GMSM implies that the result of the proposed method has fewer outliers and better local stability. The number of residues of the proposed method is second only to InSAR-BM3D and is very close to InSAR-BM3D. The reduction of residues is up to 99.97% compared with the residues of noisy image. Moreover, the computation time of the proposed method is half of the time of InSAR-BM3D. In general, the proposed method not only has outstanding filtering performance but also has high operation efficiency.

### 3.3. Experiments on Real Interferograms

**Figure 13:**
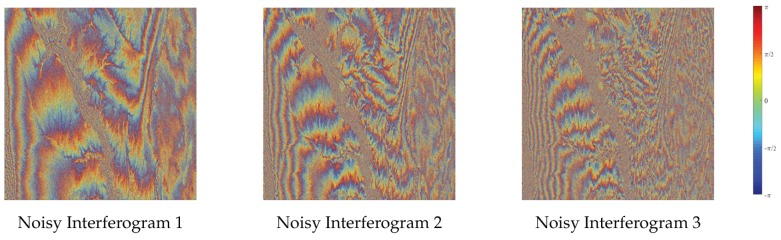
The real interferograms with different baseline (the length of baseline increase form left to right).

The original data set is three repeat-orbit InSAR data at Colorado Grand Canyon, USA, which is obtained by Alos-1 satellite. Figure 13 shows its interferograms. Baselines are 738.182, 1241.066 and 1827.02 m, respectively. The size of interferograms is 6000×5910. In noise variance estimation procedure, each interferogram is divided into 225 non-overlapping patches whose size is 400×394.

**Figure 14:**
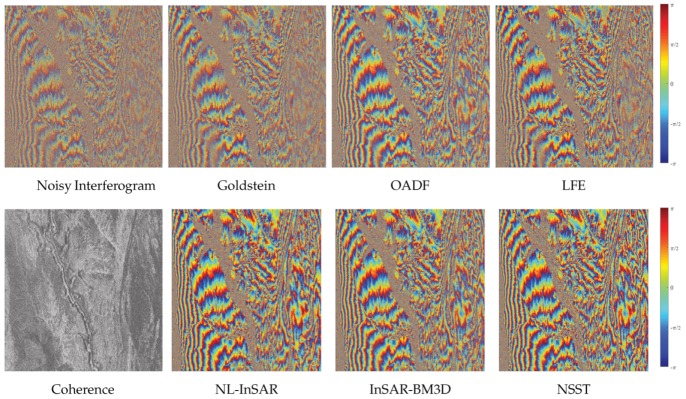
The filtered results of the real interferogram with the longest baseline.

Results are shown in Figure 14. Intuitively, the Goldstein method has completely failed. And the apparent noise remains in the result of OADF and LFE. The excellent results of NL-InSAR, InSAR-BM3D and the proposed method are similar.

**Figure 15:**
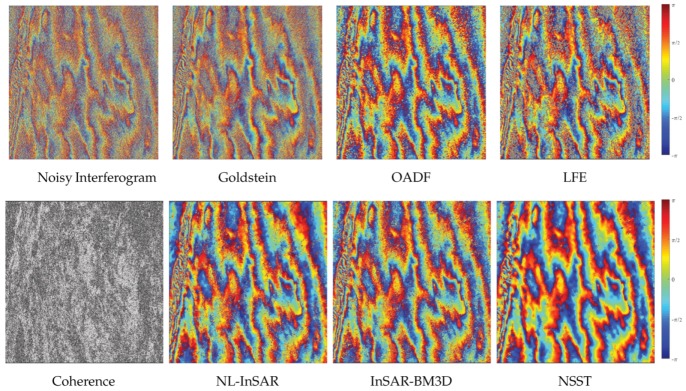
The filtered results of the low-coherence reagion (the upper right corner of real interferogram with the longest baseline (row: 1:1000, column: 4910:5910)).

In order to further compare various methods, the low-coherence region in the upper right corner (row: 1:1000, column: 4910:5910) is cropped to further analysis. Figure 15 presents denoising results of different methods. Table 7 lists the number of residues, the reduction rate of resides and computation time. Note that the bold font indicates the best performance in the table. The excellent performance of the proposed method can be confirmed directly by visual observation. In the proposed method, the reduction rate of residues (up to 99%) is remarkable and the result is more conducive for the subsequent phase unwrapping.

**Figure 16:**
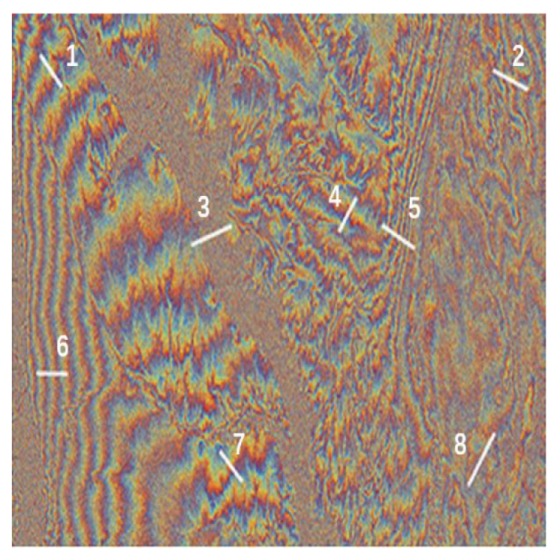
The real interferogram with the longest baseline(the order of white lines increases from left to right, from top to bottom).

**Figure 17:**
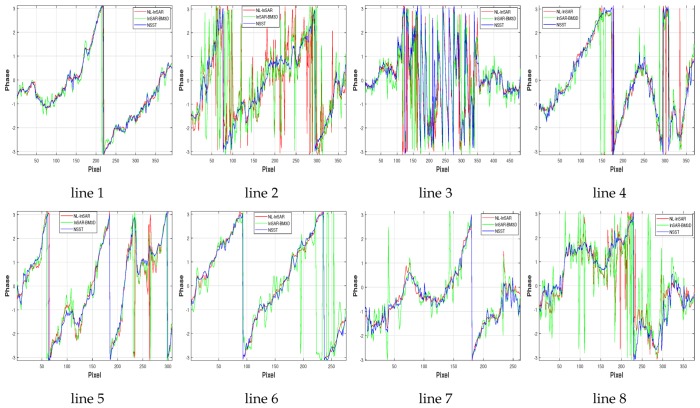
The phase profile of white lines in Figure 16 (the red, green and blue solid line represent the result of NL-InSAR, InSAR-BM3D and NSST, respectively.).

Eight phase profiles along the phase gradient direction, which involve intact phase period and satisfy local stationarity, are extracted for contrast. White lines in Figure 16 represent the phase profile at low-coherence region (line 2 and line 8), high-coherence region (line 1 and line 6), complex topography region (line 3), the region corresponding to steep topography (line 5), and so forth. As shown in Figure 17, results of phase profiles are arranged in the order of its position (increase from left to right, from top to bottom). For simplicity, Figure 17 only exhibits results of NL-InSAR, InSAR-BM3D and the proposed method, which are superior to other methods intuitively. As shown in line 3, none of three methods can recover the real phase correctly at complex topography region.The difficulty is inherent defect of interferogram with too long baseline. The phase profile at flat region with high-coherence, which corresponds to line 1 and line 6, is estimated appropriately by NL-InSAR and the proposed method. However, a few abnormal values arise in the result of InSAR-BM3D. The comparison result of the number of abnormal values at high-coherence region corresponding to steep topography (line 5) can be expressed as: the proposed method≥NL-InSAR>InSAR-BM3D. For the low-coherence region (line 2 and line 8), the proposed method outperforms NL-InSAR and InSAR-BM3D. The proposed method produces a more stationarity and authentic result. It is consistent with the result in Figure 15. On balance, the proposed method has the best comprehensive performance.

## 4. Conclusions

An attempt to the joint filtering method in multibaseline InSAR based on the statistical property of interferometric phase is proposed in this paper. This paper analyses the high-order statistical property of interferograms with different baseline and proposes an empirical assumption: the kurtosis of interferograms with different baseline keeps invariant. Simulated experiments give numerical support to it. The filtering process of the proposed method involves four parts: the NSST decomposition, the noise level estimation, pre-thresholded Wiener filter and inverse NSST. NSST gives an optimal sparse representation of distributed discontinuities, such as fringes of interferograms. We obtain a series of NSST coefficients at different scales and directions after NSST decomposition. Based on the kurtosis model in NSST domain and baseline-invariant property of interferograms, the noise variance of interferograms is represented as the solution of a constrained non-convex optimization problem. The clean NSST coefficient is estimated by the Wiener filter with the local noise variance derived by block estimation. The noise estimation experiments prove the validity of the noise level estimator. Experiments on simulated data and real data prove the edge-preservation performance and excellent filtering performance of the proposed method. Many coefficient components with the same kurtosis are obtained by NSST. Sufficient data means that the filtering performance of the proposed method is not affected by the number of interferograms. The great performance can be acquired when the number of interferograms is small. However, a large amount of memory is occupied by a large number of coefficient components. The algorithm has some requirements for memory performance. But this problem can be alleviated by adjusting the scale of NSST decomposition according to the actual computer performance.

## Figures and Tables

**Table 1:** Parameters of multibaseline interferometric synthetic aperture radar (InSAR) simulation system.

Parameters	Value
Height	642 km
Cental Frequency	3∼9.6 GHz
Bandwidth	100 MHz
Baseline	50∼500 m
Look Angle	34.5°
Baseline Orientation Angle	5°

**Table 2:** The standard deviation of kurtosis corresponding to various topography (including Cone, Building, Plain, Basin, Plateau).

**Topograpgy**	Cone	Building	Plain	Basin	Plateau
**Standard Deviation**	0.006	0.0298	0.1056	0.0442	0.0064

**Table 3:** Maximum error rate of noise level estimation.

Coherence	0.1	0.3	0.5	0.7	0.9
Actual Value	0.7044	0.6830	0.6351	0.5469	0.3632
Maximum Error Rate(%)	3.35	2.37	1.62	3.31	8.76

**Table 4:** Performance of various methods.

	MSE	Residues	Times (s)
Noisy Image	1.7897	34492	–
Goldstein	1.853	21041	**0.32**
LFE	0.7699	1454	113.39
OADF	0.8951	389	59.45
NL-InSAR	0.6577	290	459.33
InSAR-BM3D	0.6014	**0**	38.02
NSST	**0.4954**	**0**	12.68

**Table 5:** Performance of various methods.

	MSE	Residues	GMSM	Times (s)
Noisy Image	2.1571	11679	0.8297	–
Goldstein	1.9478	8490	0.8562	**0.15**
LFE	1.5504	4660	0.8975	39.00
OADF	1.4209	317	0.8684	20.95
NL-InSAR	1.3339	1211	0.8994	96.66
InSAR-BM3D	1.0631	14	0.9103	10.59
NSST	**0.9841**	**9**	**0.9343**	5.07

**Table 6:** Performance of various methods.

	MSE	Residues	GMSM	Times (s)
Noisy Image	2.1870	518970	0.6876	–
Goldstein	1.9655	371334	0.7534	**4.30**
LFE	1.5598	199348	0.8284	2784.72
OADF	1.4402	9138	0.7828	901.24
NL-InSAR	1.2885	18963	0.8648	5491.40
InSAR-BM3D	1.0564	**126**	0.9026	543.13
NSST	**1.0386**	148	**0.9213**	272.79

**Table 7:** Performance of various methods.

	Residues	Residues Reduction Rate	Times(s)
Noisy Image	174198	–	–
Goldstein	124397	28.59	**0.9**
LFE	60714	65.15	720.00
OADF	10899	93.74	433.47
NL-InSAR	15866	90.89	2542.23
InSAR-BM3D	16672	90.42	171.69
NSST	**1374**	**99.21**	91.57
